# Two decades of robotic pediatric urology: a single-surgeon evolution from adoption to excellence

**DOI:** 10.1007/s11701-026-03459-6

**Published:** 2026-05-13

**Authors:** Mohan S. Gundeti, Daniel Fu, Parviz Hajiyev, Priyank Yadav

**Affiliations:** Department of Surgery, Section of Urology, The University of Chicago Medicine, Comer Children’s Hospital, Chicago, IL USA

**Keywords:** Robotic surgery, Pediatric urology, Center of excellence, Minimally invasive surgery, Surgical training, Telesurgery, Health equity, Artificial intelligence

## Abstract

The establishment of a Center of Excellence in robotic pediatric urology is a deliberate, phased evolution—from individual pioneering procedures to an institution capable of achieving sustained long-term outcomes at par with established standards, training surgeons globally, and driving innovation. This article chronicles a single-surgeon, two-decade journey at the University of Chicago Medicine Comer Children’s Hospital across five phases: foundation and initial implementation with critical adult-pediatric partnership; program expansion, care standardization, and infant application; maturation into a Center of Clinical Excellence with durable outcomes; emergence as a training and regional hub through a mini-fellowship, proctorship program, live surgical demonstrations, and multi-institutional collaboration with the Pediatric Urology Robotic Surgery (PURS) group; and a future vision centered on global health equity, telesurgery, and artificial intelligence. This narrative serves as a framework for programs on a similar trajectory.

## Introduction

The history of urological reconstruction in children has been shaped by the relentless pursuit of outcomes that prevents unnecessary morbidity. For decades, open surgery remained the gold standard for complex congenital anomalies—augmentation cystoplasty, continent catheterizable channel creation, and ureteral reimplantation. While effective, open reconstruction carried inherent burdens: prolonged hospitalization, significant postoperative pain, the morbidity of a large abdominal incision, and an extended recovery that in many pediatric patients meant weeks away from school and normal life. The laparoscopic era offered a partially ameliorating alternative but introduced its own limitations—a steep learning curve, two-dimensional visualization, limited instrument articulation, and technical demands that made complex intracorporeal suturing difficult and procedural adoption inconsistent.

**Fig. 1:**
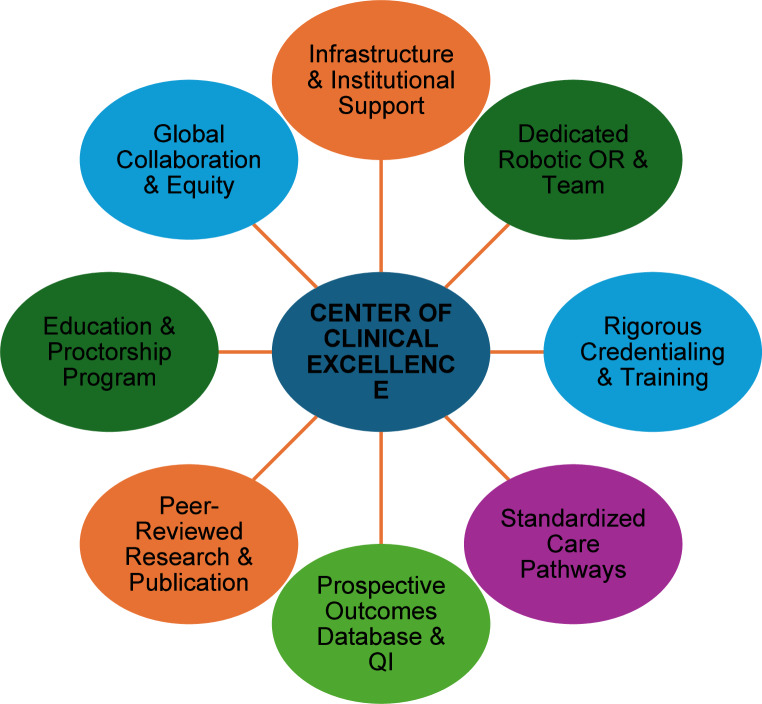
Pillars of a robotic pediatric urology center of clinical excellence. each pillar represents a domain of sustained investment essential to the development and maintenance of a center of excellence

The robotic platform was therefore not simply a procedural visa into minimally invasive surgery; it represented a genuine technological leap that addressed the specific shortcomings of laparoscopy. The three-dimensional high-definition magnification, seven degrees of instrument freedom, tremor filtration, and the ergonomic stability of the da Vinci console offered the potential for precision and reproducibility previously attainable only in open surgery—combined with the recovery advantages of minimally invasive approaches. When I joined the University of Chicago in 2007 as Director of Pediatric Urology, this was the conviction on which our program was founded: that robotic surgery could offer superior outcomes in terms of recovery, pain, and early convalescence, while preserving the functional excellence of open reconstruction. The trajectory that followed is described here (Table 1; Fig. 1).

**Table 1:** Phases of program development: university of chicago pediatric robotic urology program

Phase	Period	Primary Focus	Key Milestones	Outcomes/Evidence
I Foundation	*2007–2010*	Infrastructure, adult-pediatric partnership, team training, credentialing	World’s first RALIMA (2008); first pediatric robotic cases in Chicago; infant RALP program launched 2010	Feasibility confirmed; shorter LOS vs. open; RALIMA reported in Urology 2008(1); infant robotic-vs-open pyeloplasty data published(2)
II Expansion	*2011–2015*	Procedural expansion, care pathway standardization, QI database	LUAA technique; RALMA; dedicated OR allocation; UChicago Annual Robotic Course launched 2011	Equivalent/superior outcomes vs. open; LUAA trifecta outcomes(3); comparative pyeloplasty data(4)
III Excellence	*2015–2020*	Sustained long-term outcomes at par with literature; research and innovation	15-yr pyeloplasty mastery; largest single-institution infant RALP series; multi-institutional RALUR-EV	96% pyeloplasty success; 91.2% bilateral VUR resolution; RALIMA outcomes consistent with peer series(5,6)
IV Training Hub	*2012–Present*	Mini-fellowship, proctorship, live surgery (domestic & international), PURS collaboration	CME mini-fellowship (41 fellows, 11 countries); NARUS Co-Director since 2016; UChicago live cases; PURS studies	Multi-institutional RALMA 92% continence(6); PURS 90-day safety across 880 cases/14 institutions(7)
V Future	*2024+*	Global health equity, telesurgery, AI/ML integration	Remote robotic proctoring platform; ML hydronephrosis grading pilot; low-LMIC outreach	AI pilot published(8); telesurgery infrastructure in development; equity framework underway

AI/ML = artificial intelligence/machine learning; CME = continuing medical education; EV = extravesical; LMIC = low- and middle-income countries; LOS = length of stay; LUAA = laparoscopic ureteral advancement anastomosis; NARUS = North American Robotic Urology Symposium; OR = operating room; PURS = Pediatric Urology Robotic Surgery (collaborative); QI = quality improvement; RALIMA = robot-assisted laparoscopic ileal Mitrofanoff appendicovesicostomy; RALMA = robot-assisted laparoscopic Mitrofanoff appendicovesicostomy; RALP = robot-assisted laparoscopic pyeloplasty; RALUR = robot-assisted laparoscopic ureteric reimplantation; VUR = vesicoureteric reflux

## Phase I: Foundation and initial implementation (2007–2010)

Building a robotic program begins not in the operating room but in infrastructure, culture, and team formation. Securing institutional commitment for the da Vinci platform, establishing credentialing pathways—at a time when no standardized framework existed for pediatric robotic urology—and assembling a cohesive multidisciplinary team were the essential first acts. In the absence of any established credentialing framework specific to pediatric robotic urology, the collaboration of our adult urological colleagues proved indispensable. The mature adult program at the University of Chicago provided critical institutional scaffolding: contributing to OR setup, instrument familiarity, and robotic team culture in the earliest cases. This adult–pediatric partnership, documented in our foundational publications on the RALIMA technique and on program setup [1–10], reflected a pragmatic and collegial model for launching a pediatric robotic program within an institution where adult robotic expertise already existed. It also underscored the principle—which would later inform our own teaching—that the transfer of robotic skills across domains is both feasible and beneficial when structured mentorship is in place.

Our earliest cases focused on robotic-assisted laparoscopic pyeloplasty (RALP) for ureteropelvic junction obstruction, demonstrating outcomes equivalent to open repair with shorter hospitalization and reduced analgesic requirements [4, 11]. In 2008, our team performed the world’s first robotic-assisted laparoscopic augmentation ileocystoplasty and Mitrofanoff appendicovesicostomy (RALIMA) in a pediatric patient [1]. This completely intracorporeal procedure—until then considered beyond the realistic scope of robotic assistance—established our program’s identity as a pioneer in reconstructive pediatric robotic urology. Feasibility and initial outcomes were subsequently reported in 2011 [9], confirming shorter hospitalization, satisfactory continence, and stomal patency versus published open series.

From 2010, we extended our robotic program to include infants, deliberately pushing the limits of patient size and anatomical complexity. Performing robotic pyeloplasty in patients weighing less than 8 kg demanded significant adaptation of technique—port placement, instrument management, and pneumoperitoneum settings had all to be re-evaluated for the unique physiological and anatomical constraints of infant surgery. Our initial comparative analysis of robotic versus open pyeloplasty in infants, published in 2013, provided one of the earliest institutional validations that the robotic approach was feasible and safe in this smallest of patient populations, with comparable outcomes to open repair and similar length of hospital stay [2]. This early commitment to infant robotic surgery distinguished our program and laid the groundwork for what would become the largest single-institution infant RALP series in the literature [12].

### Phase II: Program expansion and standardization (2011–2015)

Phase II was characterized by deliberate expansion of our procedural portfolio alongside rigorous technique refinement. At the time, the literature on robotic ureteral reimplantation was characterized by heterogeneous technique descriptions, variable outcome reporting, and conflicting data on efficacy and complications for both laparoscopic and robotic approaches. There was a demonstrable inadequacy of standardized evidence for both modalities. As we expanded our case volume, our commitment was to prospectively capture and publish our outcomes as we standardized our techniques, contributing to a literature in genuine need of high-quality, single-surgeon and multi-institutional data.

Our development of the LUAA technique for robotic extravesical ureteral reimplantation reflected a philosophy of fundamental anatomical re-examination rather than incremental modification of the existing approach. Rather than adapting the established open extravesical technique with minor robotic adjustments, we revisited the entire conceptual basis of ureteral reimplantation—specifically, the orientation of autonomic nerve fibers at the base of the bladder and the trigone. The risk of secondary neurogenic bladder dysfunction following bilateral extravesical reimplantation had been attributed to inadvertent disruption of these perivesical nerve plexuses. The LUAA acronym—Length of detrusor tunnel, U-stitch, Adventitial inclusion, Apical alignment suture—embeds an anatomically informed nerve-sparing framework into each technical step, with the explicit goal of reducing the risk of postoperative neurogenic bladder and optimizing the trifecta of outcomes: reflux resolution, absence of retention, and freedom from perioperative complications [3]. Our decade-long data validated this approach with a 96.7% VUR resolution rate and low retention rates in bilateral cases [3].

Across all procedures, we maintained a discipline of continuous video review of our operative recordings, systematic analysis of outcomes data, and iterative technique refinement. This culture of self-audit and critical re-evaluation—not merely reporting results but interrogating them—was central to how the program matured. A dedicated robotic surgery database, introduced during this phase, enabled prospective capture of operative metrics, complications, and functional outcomes, providing the infrastructure for all subsequent research and quality improvement.

### Challenges, adverse events, and lessons learned

No program of this complexity evolves without encountering significant obstacles, and honesty about those obstacles is as instructive as the achievements described above. Several challenges shaped the program’s development in ways directly relevant to institutions seeking to replicate this model. The program’s integration within the Section of Urology meant that team consistency had to be deliberately engineered around a fixed surgical and nursing core, although it also meant that the initiation problems like equipment acquisition as well as surgical assistance were not an issue with this program. Anesthetic management of children undergoing prolonged robotic procedures demanded ongoing protocol development.

The most consequential adverse event in the program’s early phase—a vascular injury attributable to inadvertent instrument over-advancement during port introduction—triggered a formal root cause analysis, a two-month program suspension, mandatory team retraining, and a structural redesign of the program’s operational model. Challenges relating to OR block time allocation, patient volume and referral network development, resident training equity, operative time and productivity pressures, and institutional economic sustainability were equally formative. Rather than obstacles that were simply overcome, these were experiences that shaped the program’s culture, policies, and design. Each is summarized in Table 2 alongside the mitigation strategies that proved most effective, with the intention that this account serves as a practical road map for programs in earlier stages of development.

**Table 2:** Challenges encountered during program development and mitigation strategies

Challenge	Description	Mitigation Strategy
Institutional integration and adult–pediatric partnership	No standalone pediatric robotic infrastructure existed at program inception. Pediatric urology was nested within the Section of Urology in the Department of Surgery, without dedicated pediatric robotic OR resources, equipment, or nursing teams	Leveraged the established adult robotic program for shared OR resources, equipment familiarity, and team culture. Residents rotated from the urology program, providing a pipeline of trainees already familiar with the robotic platform in an adult context
Team building and consistency	Complex pediatric robotic cases demand a team that anticipates rather than reacts	A core team of scrub technicians, circulating nurses, and bedside assistants was kept consistent across all cases. The primary surgeon was present in the room from skin incision to closure throughout every case. Residents were integrated as supervised trainees, not as substitutes for core team members. A second fully trained team was subsequently developed to ensure continuity
Pediatric anesthetic complexity	Infrared light from early-generation camera systems caused intraoperative hyperthermia in small patients. Prolonged pneumoperitoneum and Trendelenburg positioning imposed additional physiological demands. Diaphragmatic excursion during intracorporeal suturing required real-time ventilator adjustments, and coordination with the anesthesia team was not always intuitive	Bair Hugger placed but not inflated unless body temperature required it. End-tidal CO₂ targets and tidal volume protocols developed collaboratively with the anesthesia team for each procedure type. Diaphragmatic movement during suturing managed through explicit real-time communication between surgeon and anesthetist. The primary surgeon assumed the designated communication role throughout each case
Intraoperative communication and team coordination	Complex pediatric robotic cases require constant real-time communication across the console surgeon, bedside assistant, scrub technician, circulating nurse, and anesthetist. Without a designated coordinator, critical information risks being missed or acted upon too late	The primary surgeon assumed full liaison and communication responsibility throughout each case, regardless of console position. Standardized intraoperative briefings, structured time-outs, and explicit role assignments were introduced early and maintained consistently as team composition evolved
Handling adverse events:	A vascular injury occurred in the program’s early phase due to inadvertent instrument over-advancement during port introduction. The event necessitated immediate open conversion and triggered a full program review	Formal root cause analysis conducted. Program suspended for two months; all team members underwent mandatory retraining in pediatric robotic instrument handling and port introduction protocols. Critical structural insight: a single operative team is insufficient — a second fully trained team was established to ensure patient safety in the event of any team member’s unavailability. A near-miss and adverse event reporting culture was formalized
	A needle was retained in the abdominal cavity in a case that lasted more than 8 h. The nursing team changed during the surgery and counts were mentioned as correct. However, postoperative Xray found a needle in the abdomen	The incidence was discussed with the family with full disclosure. The family chose to observe. No adverse consequence happened during more than 10 years of follow up. We mandated X-ray at the end of the surgery for all cases lasting more than 8 h and also noted all needle counts on board as staff handovers could be unreliable with extra-long cases
Resident training and equity of experience	Rotating urology residents had variable robotic familiarity and limited pediatric exposure. Exclusion from meaningful console time could generate resentment and undermine the program’s credibility as a training environment. Unstructured resident involvement risked both patient safety and inadequate supervision	Structured prerequisite pathway introduced: dry lab simulation and adult robotic experience required before any pediatric console time. Initial pediatric cases completed with residents in bedside assistant role only. Console time introduced incrementally for procedural steps appropriate to each resident’s level. Time savings accrued as the primary surgeon’s proficiency improved were explicitly reallocated to supervised resident training
Operative time, productivity pressure, and anesthesia risk	Early complex cases required significantly extended operative times, creating tension with OR scheduling efficiency, institutional RVU productivity expectations, and anesthesia risk for small patients from prolonged general anesthesia and pneumoperitoneum	Productivity benchmarks set relative to the surgeon’s own learning curve rather than adult robotic or national benchmarks. Proficiency-related time savings explicitly reinvested in resident training once mastery was achieved. Additional anesthesia time for resident training weighed explicitly against patient risk at each case—structured judgment, not routine policy
Operating room block time allocation	Consistent OR block time is rarely available at program inception. Cases must be scheduled opportunistically in open slots, which may result in unpredictable timing, suboptimal team availability, and fragmented case accumulation—slowing learning curve progression for both surgeon and team	Case volume built incrementally using available open time in the early phase. Safety and efficiency data presented systematically to the OR committee to justify dedicated block time allocation. Adult urology block time leveraged in the early phase where scheduling overlap allowed. Block time was secured progressively as the program demonstrated consistent volume and quality
Patient volume and referral network development	Insufficient case volume in the early program phase limits learning curve progression, reduces team familiarity, and creates financial pressure. Pediatric complex reconstructive cases are inherently low-frequency and require a broad referral catchment area	Expanded clinic coverage including offsite and satellite visits established to increase geographic reach. Proactive networking with referring pediatric urologists, nephrologists, neonatologists, and primary care pediatricians. Educational outreach to community providers to raise awareness of robotic options for complex pediatric urological conditions. Academic visibility through publications and conference presentations enhanced institutional reputation and referral confidence
Institutional economics and program sustainability	Robotic surgery programs involve substantial capital investment (platform acquisition and maintenance), recurring disposable costs per case, and relatively unfavorable per-case RVU yield for complex reconstructive procedures. Justifying program economics to hospital administration—particularly under conditions of reduced government health funding—is a persistent challenge that intensifies in the program’s early phase before volume and reputation are established	Cost-sharing with the established adult robotic program reduced per-case equipment overhead in the early phase. Research grant funding secured to support innovation and outcomes work, reducing dependence on clinical revenue alone. Institutional value argument articulated in terms of downstream referral generation, trainee recruitment, and reputational benefit rather than per-case margin alone. Transparent engagement with hospital administration around the distinction between loss-leader investment cases (complex reconstruction) and higher-volume procedures (pyeloplasty, reimplantation) that sustain program economics
Mini-fellowship development	Dedicated ARC Lab, robot and staff for the program were unavailable in the initial phase of development	ARC Lab, robot and inventory were acquired from the University of Chicago and centralized training facility; ad-hoc help from residents and fellows; constant supervision of the primary surgeon for both ARC Lab and lectures while keeping the regular work schedule in order to ensure safety

RVU = relative value unit; OR = operating room; ARC = Advanced Robotics and Control

### Phase III: Center of clinical excellence (2015–2020)

By mid-program, the University of Chicago had accumulated the volume, experience, and technical refinement to sustain long-term outcomes at par with those reported in the established literature across a portfolio of complex procedures—defining the threshold of a true Center of Clinical Excellence.

**Fig. 2:**
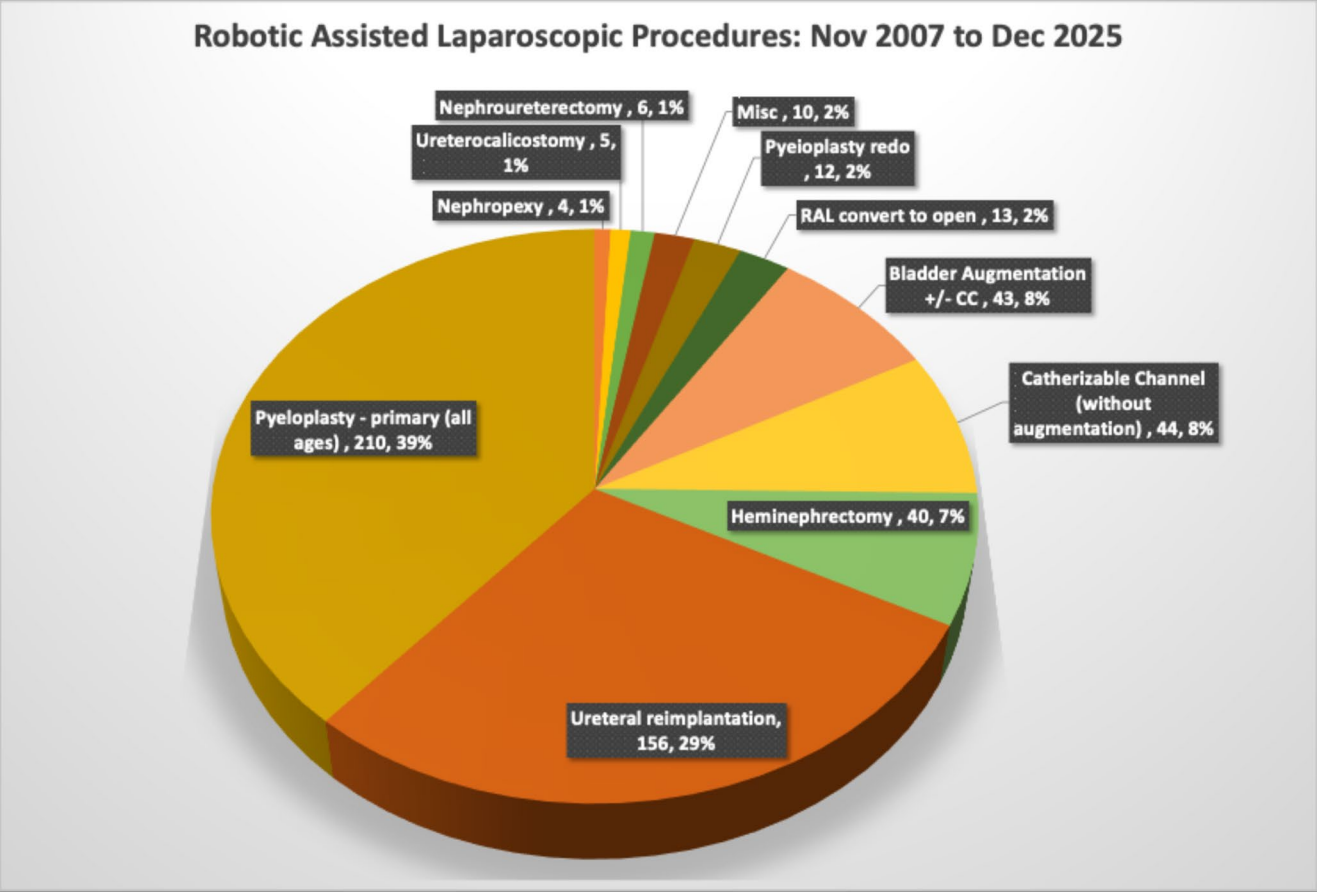
Pie-chart showing the distribution of different robotic procedures performed between 2007 and 2025 at our center (*n* = 543)

In pyeloplasty, a 15-year single-surgeon series published in 2025 defined mastery over the full learning curve with an overall success rate exceeding 96% and progressively declining operative times [13]. For infant pyeloplasty, our series of 44 consecutive patients below 12 months demonstrated 100% technical success with mean blood loss of 7 mL and median hospitalization of one day [12]—outcomes consistent with the multi-institutional infant RALP collaborative [2] and with the multi-center laparoscopic versus robotic infant pyeloplasty comparison published in the literature [14]. For bilateral RALUR using the LUAA technique, our VUR resolution rate compares favorably with the prospective multicenter RALUR-EV study [15] and with the review by Essamoud et al. [16]. For RALIMA, our 15-year comparative series demonstrated perioperative and functional outcomes at par with the best published open series [8], with continence rates consistent with the multicenter RALMA data [6]. Comparative series from peer institutions [17, 18] have independently reported high continence rates, affirming that expert robotic reconstruction delivers durable results equivalent to the open standard across institutions. A list of all robotic procedures performed at our center is presented in Fig. 2.

Research and innovation were equally defining features of this phase. Our multi-institutional ureterocalicostomy cohort addressed technically complex upper-tract anomalies seldom covered in robotic series [19]. Our study on health equity in access to robotic surgery found that demographic factors were associated with differential access to minimally invasive procedures [20]—findings that directly informed our subsequent advocacy for equity-centered program design.

### Phase IV: Training center and regional hub (2012–Present)

**Fig. 3:**
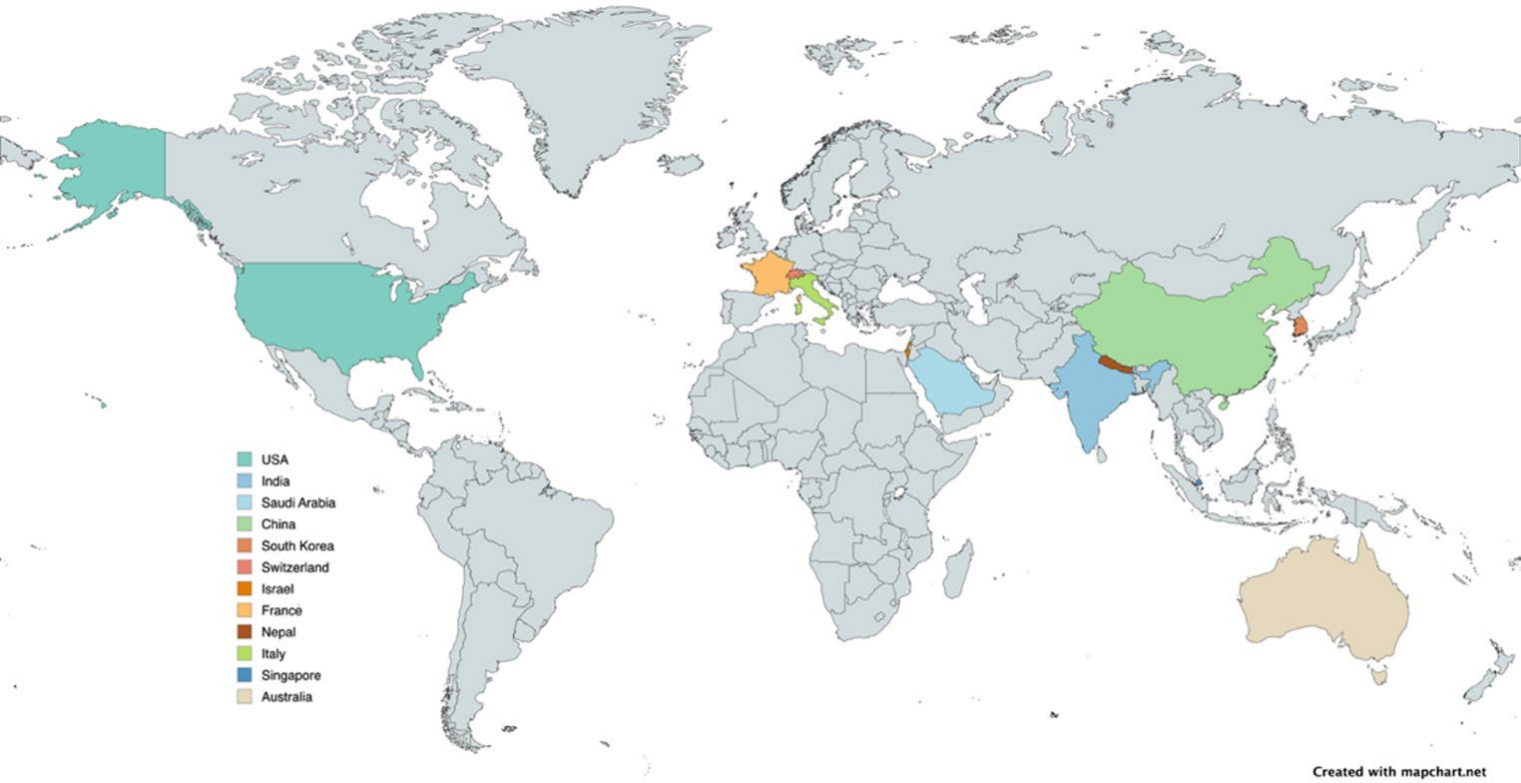
World map showing the outreach of the robotic surgery mini-fellowship program

Clinical excellence confined to a single institution has a limited radius of benefit. The Pediatric Robotic Surgery Mini-Fellowship, established at the University of Chicago in 2012, was our principal response to that constraint. This intensive five-day CME-accredited course—structured in two modules covering upper and lower urinary tract robotic surgery, with a one-to-one faculty-to-attendee ratio, hands-on simulation, live case observation, and video review—was designed specifically for practicing surgeons rather than trainees. To date, the program has trained 41 fellows from 11 different countries, making it one of the most internationally diverse dedicated pediatric robotic surgery training programs in the world. A formal evaluation published in 2019, reporting outcomes from the first 29 international participants, demonstrated that the program successfully enabled surgeons to advance to robotic pyeloplasty, ureteral reimplantation, and reconstructive cases in their home institutions (Fig. 3) [21].

Complementing the mini-fellowship, a structured visiting surgeon and proctorship program provided graduated in-person and remote guidance to surgeons during their early independent cases. Evidence confirms that proctored training shortens the robotic pyeloplasty learning curve and improves early outcomes [22].

Within the United States, live surgical demonstrations have been a sustained vehicle for domestic educational outreach. The University of Chicago Annual Robotic Urology Course—running continuously since 2011—has provided practicing urologists with moderated live surgical cases for continuing medical education, covering the full spectrum of robotic pediatric procedures from pyeloplasty to complex bladder reconstruction. At the North American Robotic Urology Symposium (NARUS), where I have served as Co-Director since 2016, live cases have exposed national audiences to complex robotic reconstructive procedures in real time, with interactive faculty commentary. The ethics, patient safety protocols, and clinical outcomes underpinning our institutional live-case program have been formally described in a dedicated publication [23], providing a transparent and reproducible framework that other centers can adopt. Internationally, our live surgical demonstration program has reached more than 30 countries across six continents, including Chile, Mexico, Spain, Israel, India, Australia, and Singapore. A summary of all our live surgical demonstrations is presented in Table 3.

**Table 3:** Summary of live robot-assisted pediatric urological procedures from 2011 through 2024 (total cases = 57)

Procedure	*n*	Age (years)	Indication/Grade	OT (min)	LOS (days)	Intraop Complications	30-day CDG III Complications
RAL-P	18	8.2 ± 4.8 (13 infants)	SFU IV/III: 13/5	117 ± 28	NR	0	1 (hematoma)
RALUR	17	5.9 ± 4.9	VUR III/IV/V: 10/5/3	103.8 ± 11.9	1.8 ± 0.6	0	1 (retention, bilateral case)
RAL-HN	8	5.8 ± 3.2	Duplex system	154 ± 35	3.5 ± 4.3	0	1 (hematoma)
RALMA	4	13.4 ± 5.2	Neurogenic bladder	109 ± 10.9	4.5 ± 2.4	0	0
RALIMA	1	8	Complex bladder dysfunction	345	7	0	0
Robotic BNR + APV	1	17	Bladder outlet incompetence	360	32	0	1 (bowel injury)
RAL Excision of Prostatic Utricle	5	3.7 ± 5	Utricle	96.8 ± 12.5	3.8 ± 1.2	0	0
Robotic Vaginal Pull-through	1	3	Müllerian anomaly	150	5	0	0
RAL Nephrectomy	1	1.8	MCDK	80	2	0	0
RAL Ureteroureterostomy	1	6.5	Duplex system	100	NR	0	0

BNR + APV = Bladder neck reconstruction with appendicovesicostomy; CDG III = Clavien–Dindo Grade III complications; LOS = Length of stay; NR = Not reported; OT = Operating time; RAL-HN = Robot-assisted heminephrectomy; RAL-P = Robot-assisted laparoscopic pyeloplasty; RALIMA = Ileal Mitrofanoff appendicovesicostomy; RALMA = Mitrofanoff appendicovesicostomy; RALUR = Robot-assisted laparoscopic ureteric reimplantation; SFU = Society for Fetal Urology grade; VUR = Vesicoureteric reflux

Multi-institutional collaboration through the Pediatric Urology Robotic Surgery (PURS) group formalized the research dimension of our hub role. The PURS consortium—bringing together pediatric urologists from leading North American centers including Children’s Hospital of Pittsburgh, Seattle Children’s, Texas Children’s, Emory, Johns Hopkins, and others—generated foundational safety and outcome data for the field. The 90-day perioperative complication study by Dangle et al., encompassing 880 pediatric robotic cases across 8 institutions, established that the overall complication profile of pediatric robotic procedures was comparable to equivalent open and laparoscopic approaches [7]. The prospective multicenter RALUR-EV study, with our institution as the coordinating center, provided the most robust comparative evidence to date for robotic ureteral reimplantation [15]. These PURS collaborations ensured that our institutional techniques were tested and validated in the broader multi-center context.

### Phase V: Future directions

At the foundation of our future vision lies the principle of health equity. The children who stand to benefit most from expert robotic pediatric urological surgery are often those with the least access to it. In high-income countries, access inequities driven by race, insurance status, and geography already exist within different institutions [20]. Globally, the disparity is far more pronounced: children in low- and middle-income countries lack access not only to robotic platforms but to the trained surgeons, institutional infrastructure, and follow-up systems required to deliver complex reconstructive urological care safely. Our program’s educational endeavors—the mini-fellowship, proctoring, and live surgery teaching in 30 + countries—represent a deliberate effort to close this gap. The next frontier is extending this reach through technology.

Telesurgery and remote robotic proctoring offer the most promising near-term mechanism for democratizing surgical expertise. Telementoring—whereby an expert provides real-time verbal and visual guidance to a surgeon at a remote site—has been demonstrated to be safe and effective across surgical disciplines, with evidence that it can accelerate skill acquisition and can potentially improve early outcomes [24]. We are developing the infrastructure for remote robotic proctoring as an extension of our existing proctorship program, integrating secure bidirectional video platforms and, in future iterations, telestration tools that allow the remote proctor to annotate the surgical field in real time. The capacity to transmit expertise across borders has the potential to fundamentally alter the geography of high-quality pediatric urological care.

The integration of artificial intelligence (AI) and machine learning (ML) represents the second pillar of our future vision. Our pilot study of ML for distinguishing high- and low-grade hydronephrosis on ultrasound established proof-of-concept for AI-assisted diagnostic standardization [8]. Looking ahead, AI-assisted surgical planning—incorporating three-dimensional anatomical reconstruction, quantitative renal function assessment, and predictive outcome modeling—has the potential to support decision-making in complex cases, particularly for surgeons in lower-resource settings. Our 15-year prospective database represents an invaluable resource for training and validating such models, ultimately aiming at prevention of renal failure in children.

## Conclusion

The evolution of our program from a newly formed division performing its first robotic procedures to a globally recognized Center of Excellence is a story of phased, deliberate building—of infrastructure, technique, evidence, educational reach and honest reckoning with the obstacles encountered along the way. We believe the phased framework described here is reproducible at institutions of varying size and resource level, provided the foundational investments in team training, dedicated OR allocation, and prospective outcome tracking are made before volume expansion is attempted. The specific procedures and technologies will differ across settings; the underlying principles—disciplined phasing, rigorous outcome measurement, anatomically grounded technique development, commitment to education, and attention to equity—are universal. The honest sharing of outcomes allows critical assessment of this technological application to the vulnerable pediatric population. As robotic surgery, telesurgery, and AI continue to converge, the most meaningful measure of a Center of Excellence will be not only what it achieves within its own walls, but what it enables beyond them.

## Data Availability

No datasets were generated or analysed during the current study.
